# A Combination of UTMD-Mediated HIF-1*α* shRNA Transfection and TAE in the Treatment of Hepatic Cancer

**DOI:** 10.1155/2019/1937460

**Published:** 2019-02-19

**Authors:** Yangying Liao, Haibo Luo, Zhizhong He, Yongpei Kuang, Peifen Chen, Xiuzhen Zhang, Junjun Chen, Qirong Wen, Yuhuan Xie, Shangwei Ding

**Affiliations:** ^1^Department of Ultrasound, Dongguan People's Hospital Affiliated to Southern Medical University, No. 3 New Valley Chung Wan Road South, Wanjiang District, Dongguan, 523059, Guangdong, China; ^2^The Third Affiliated Hospital, Center for DAMP Biology, Key Laboratory for Major Obstetrics Diseases of Guangdong Province, Key Laboratory of Protein Modification and Degradation Laboratory of Guangdong Higher Education Institutes, School of Basic Medical Sciences, Guangzhou Medical University, Guangzhou, Guangdong 510510, China; ^3^Department of Ultrasound, the First Affliated Hospital of Guangzhou Medical University, Guangzhou, Guangdong, 510120, China

## Abstract

To explore the antitumor effect of hypoxia-inducible factor-1*α* short hairpin RNA (HIF-1*α* shRNA) delivered by ultrasound targeted microbubble destruction (UTMD) and transcatheter arterial embolization (TAE) on rats with hepatic cancer. After the models of transplantation hepatoma were established, Wistar rats were randomly divided into 4 groups: Control group, UTMD group, TAE group, and UTMD+TAE group. Contrast-enhanced ultrasound (CEUS) was used to monitor tumor size on day 14 after four different treatments. Western blotting and immunohistochemistry were applied to measure the protein level of HIF-1*α* and VEGF in the hepatic cancer tissue. In comparison with UTMD+TAE group (21.25±10.68 days), the mean survival time was noticeably shorter in the Control group and TAE group (13.02±4.30 days and 15.03±7.32 days) (p<0.05, respectively). There was no statistical difference between UTMD+TAE group and UTMD group of the mean survival time (p>0.05). In addition, our results proved that the tumor sizes in UTMD+TAE group were obviously smaller than those in other groups (p<0.05, respectively). By CEUS, we clearly found that the tumor size was the smallest on day 14 in the UTMD+TAE group. The western blotting and immunohistochemistry results proved that the protein levels of HIF-1*α* and VEGF in UTMD+TAE group were obviously lower than those in TAE group and Control group on days 7 and 14 (p<0.05, respectively). However, there was no statistical difference between UTMD+TAE group and UTMD group (p>0.05). In this study we tried to explore the antitumor effect through a combination of UTMD-mediated HIF-1*α* shRNA transfection and TAE on rats with hepatic cancer. Our results showed that UTMD-mediated HIF-1*α* shRNA transfection and TAE can obviously silence HIF-1*α* and VEGF expression, thereby successfully inhibiting the growth of the tumor.

## 1. Introduction

Transcatheter arterial chemoembolization (TACE) is a widely used palliative treatment for patients with nonsurgical hepatocellular carcinoma (HCC). After blocking the blood supply with TAE, cancer cells are in an intense state, lacking the necessary oxygen, which causes some to appear necrotic. Those that do survive will be in a high expression of HIF-1*α*, causing tumor recurrence and metastasis after initial treatment. Under the condition of hypoxia, HIF-1*α*, which can stimulate the formation of new blood vessels and alleviate the cell hypoxia, can be higher in hepatoma cells and increase the survival of cancer cells [1, 2]. Current research shows that HIF-1*α* plays an important role in signal transduction pathway of VEGF under hypoxic condition. It can increase gene expression and enhance protein translation for VEGF. In the process of gene therapy, the interference gene can effectively silence HIF-1*α* expression in liver cancer cells under the condition of hypoxia, so as to effectively restrain the formation of new blood vessels in liver cancer tissue after TAE. Then due to the continue hypoxia, necrosis, and apoptosis of cancer cells, the treatment effect of TAE will be improved significantly and the recurrence of liver cancer will be greatly reduced. The combined treatment method (between TAE and gene therapy) would be a great boon for liver cancer patients. A lot of research confirms that UTMD can effectively promote gene delivery [3–9] and has the practical value of transfection in vivo at the same time [10–12]. In this study, HIF-1*α* shRNA was imported into liver cancer cells in the aid of UTMD and then embolism tumor blood vessels by TAE. Due to the lack of support for the new blood vessels and due to being in a state of continuous hypoxia, liver cancer may eventually be cured through the use of this treatment method.

## 2. Material and Method

### 2.1. Animals and Cell Line

Wistar rats (weighing 100~150g) were purchased from the Animal Experimental Center of Southern Medical University (Guangzhou, China). The experimental protocol was approved by the Institutional Animal Care and Use Committee (IACUC) of Southern Medical University. The cell line Walker 256 was kindly provided by Cell Biology Laboratory in Tongji Medical College, Huazhong Science and Technology University. Then it cultured in 1640 culture medium containing 15% fetal bovine serum and 1% penicillin and streptomycin in a humidified incubator at 5% CO2 and 95% humidified atmosphere air at 37°C.

### 2.2. Materials and Instruments

1640 culture medium (Gibco), Opti-MEM (Gibco), FBS (Gibco), penicillin, streptomycin, trypsin, RIPA, and Cell Counting Kit-8 (Gibco), rabbit anti-rat HIF-1*α* (Novus Biologicals), mouse anti-rat VEGF (Novus Biologicals), mouse anti-rat glyceraldehyde 3-phosphate dehydrogenase (GAPDH; Abcam), DNA Ladder (Solarbio, China), SonoVue (Bracco, Italy), HIF-1*α* shRNA were synthesized by the Genomics Institute, Sonitron 2000V (Nepa Gene, Japan), Microscope (Nikon, Japan), High Speed Freezing Microcentrifuge (SCILOGEX, Americ ), and Multiskan GO (Thermo Scientific, America).

### 2.3. Plasmid

Based on the previous research, we successfully constructed a gene vector plasmid RSH050798-1-HIVU6 (OS375737). It contained reporter gene (eGFP) and shRNA (with target sequences of CCATCAGTTACTTACGTGT, used to target HIF-1*α*), which can express green fluorescent protein within the cells and silence the expression of targeted HIF-1*α*. We have proved them in previous experiments.

### 2.4. Preparation of Subcutaneous Hepatic Cancer Model in Mice

Walker 256 cell suspension (5×10^5^) was injected into Wistar rats subcutaneously. After 7~10 days, neck tumor tissues was removed and separated when its volume reached nearly 1.0 cm3. The tumor tissue was divided into small pieces (about 2mm in diam). Then the divided small pieces were implanted into the central area of the left lobe of the liver (about 1.0cm far from the edge of the liver) in rats to induce HCC, following a similar path as the previously reported study [13, 14].

### 2.5. Grouping

Based on the size and the vitality of the transplanted tumor by CUES on day 12, the qualified models were left for the subsequent experiments. Then the tumor-bearing rats were randomly assigned into four groups, including Sham Control group: after opening the abdominal cavity, there was no UTMD or TAE treatment; UTMD group: only HIF-1*α* shRNA transfection induced by UTMD; TAE group: only treatment with TAE; UTMD+TAE group: a combination treatment of UTMD and TAE.

### 2.6. The Combined Treatment of UTMD and TAE

Under aseptic conditions the abdominal cavity was opened to expose the liver cancer tissue. Mix the SonoVue suspension (4*μ*L/g) with HIF-1*α* shRNA (1*μ*g/g) according to the weight of the rats. The mixture remained still for 15min at room temperature and then injected into the rats through the tail vein at a constant speed (0.5mL/min). A probe was then placed onto the liver tissue surface to start irradiation. The irradiation parameters were as follows: intensity 1.5w/cm^2^, duty cycle 20%, time 6min, and frequency 1MHz. After gene transfection was induced by UTMD, the liver, stomach, and duodenum were fully exposed before finding and identifying the structures (such as gastroduodenal artery, common hepatic artery and proper hepatic artery). The gastroduodenal artery was located and its distal artery was litigated. The suture was wrapped around the proximal gastroduodenal artery and tensed when silastic tubing was inserted from the gastroduodenal artery incision. Then iodized oil (0.2mL/kg) and saline was then injected slowly. The speed had to be slow enough to ensure that the iodized oil was delivered into the liver successfully. After the catheter was removed, the proximal gastroduodenal artery was ligated. To finish the procedure, the common hepatic artery should be loosened and intrahepatic arterial blood supply should be restored. After HIF-1*α* shRNA transfection induced by UTMD, the liver cancer blood vessels were embolized by TAE, then the abdominal cavity was closed.

### 2.7. Assessment of Therapeutic Effect

Under the four therapeutic methods, the rats were used to evaluate the survival time during the following 28 days. The hepatoma tissue was monitored by CEUS to compare the change of tumor size among the four groups. At the same time, the rats that survived more than 14 days were used for statistical analysis of the tumor size (V(mm^3^)=(length×width×height×л/6)) [15]. And the hepatoma tissue was also used for western blotting and immunohistochemical staining.

### 2.8. Immunohistochemical Staining

Following a standard protocol, hepatoma tissue was used for immunostaining of HIF-1*α* and VEGF on day 14. After deparaffinization and rehydration, slides were treated with antigen retrieval at 97°C for 45 minutes. Sections were then stained with primary antibodies (diluted in 1:100) and corresponding secondary antibodies (diluted in 1:5000) following the Catalyzed Signal Amplification System. Nuclei were counterstained with hematoxylin. Two or three fields from each slide were counted to determine the staining frequency of HIF-1*α* and VEGF.

### 2.9. Western Blotting

3, 7, and 14 days after transfection, HIF-1*α* and VEGF proteins were then extracted by the corresponding protein extraction reagent and preserved at -80°C; following a standard protocol, 40ml/L concentrated gel, 100ml/L separation gel, prestained protein Marker 3.0*μ*L, and 20*μ*g/well sample protein. A sample of 100mL/L SDS-PAGE was added before being put through electrophoresis at 60V. After 30min, it was changed to 100V and stopped when bromophenol blue ran to the bottom. The protein was synchronously transferred to PVDF membrane at 20V for 50min. For 4h the membrane was blocked with 50mL 5% skim milk in tris-buffered saline (TBST) at room temperature. Primary antibody was then added followed by incubation for 2h at room temperature and overnight incubation at 4°C. After washing the membrane, the appropriate concentration of secondary antibody with HRP for incubation was added at 37°C for 2h. Finally, after washing the membrane, chemiluminescence was detected. The protein band was visualized and quantified with Software Quantity One. The relative density of each band was determined by the ratio to that of the internal control, GAPDH.

### 2.10. Statistical Analysis

Data was expressed as mean±standard deviation ($\overset{-}{x}\pm s$). Student's t-test was used to analyze experimental data between two groups. The survival was examined via the Kaplan-Meier method with a log rank test between two groups. P<0.05 was considered statistically significant. Analyses were performed by using the SPSS software version 19.

## 3. Results

### 3.1. The Survival Curve and Tumor Growth

**Figure 1:**
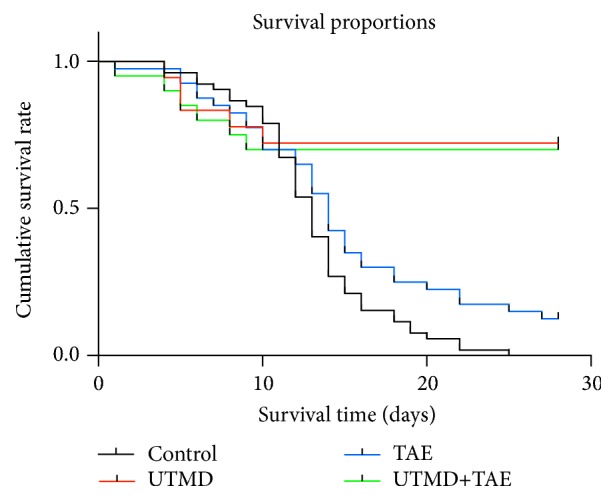
The survival curve of hepatic cancer rats in four groups.

Except for those that were executed on a regular basis, anesthetized on accident, and were failures regarding the experiment, we observed the remaining rats for 28 days and record their survival time. The death rate reached 100% (42/42) in the Control group on the 28th day, but it was 87.5% (35/40) in TAE group. After effective treatment, the death rate has dropped into 27.8% (10/36) in UTMD group and 30.0% (12/40) in UTMD+TAE group. The mean survival time in UTMD+TAE group and UTMD group was 21.25±10.68 days versus 22.00±10.03 days, with no statistical difference between the two groups (p>0.05). In comparison with UTMD+TAE group, the mean survival time was obviously shorter in the Control group and TAE group (13.02±4.30 days and 15.03±7.32 days) (p<0.05, respectively) (Figure 1).

**Figure 2:**
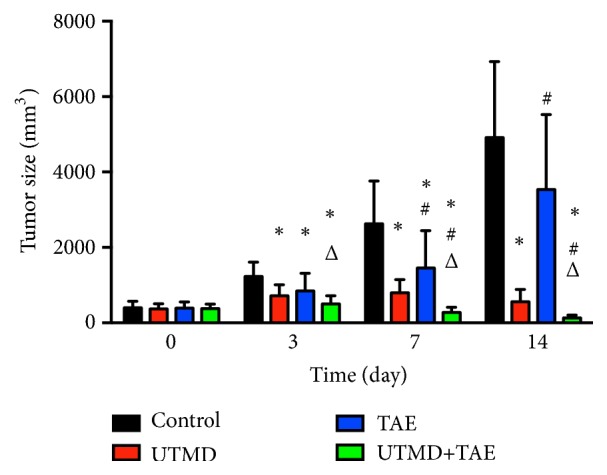
The change of tumor size in different groups. A: Control group; B: UTMD group; C: TAE group; D: UTMD+TAE group. The comparison between two groups at the same time, *∗* p<0.05: comparison with control group; #p<0.05: comparison with UTMD group; Δp<0.05: comparison with TAE group.

**Figure 3:**
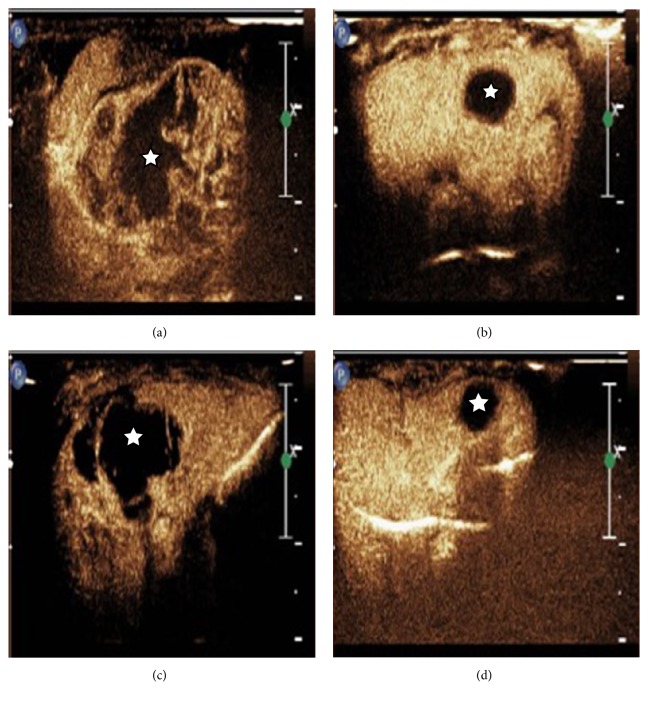
The change of tumor size by CEUS in four groups at day 14. (a) Control group; (b) UTMD group; (c) TAE group; (d) UTMD+TAE group. ☆ indicates the location of the tumor.

The rats that survived more than 14 days were used for tumor size analysis. On days 7 and 14, tumor size was significantly smaller in UTMD+TAE group (with a size of 273.4±136.5 mm^3^ and 132.3±75.7 mm^3^) compared with the control group (2625.4±1136.8 mm^3^ and 4913.1±2014.9 mm^3^), with UTMD group (796.0±349.3 mm^3^ and 559.3±329.1 mm^3^), with TAE group (1455.8±987.1 mm^3^ and 3534.7±1987.1 mm^3^) (p<0.05, respectively) (Figure 2). Meanwhile, it showed that the tumor size was the smallest on day 14 in the UTMD+TAE group by CEUS in Figure 3.

### 3.2. The Protein Expression of HIF-1*α* and VEGF in Hepatoma Tissue

**Figure 4:**
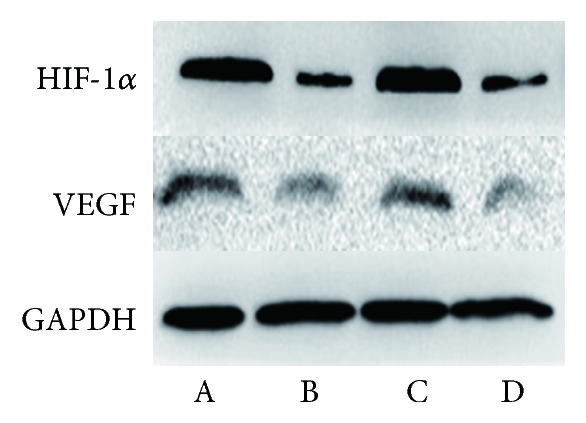
The relative expression of HIF-1*α* and VEGF in four groups at day 14. A: Control group; B: UTMD group; C: TAE group; D: UTMD+TAE group.

**Figure 5:**
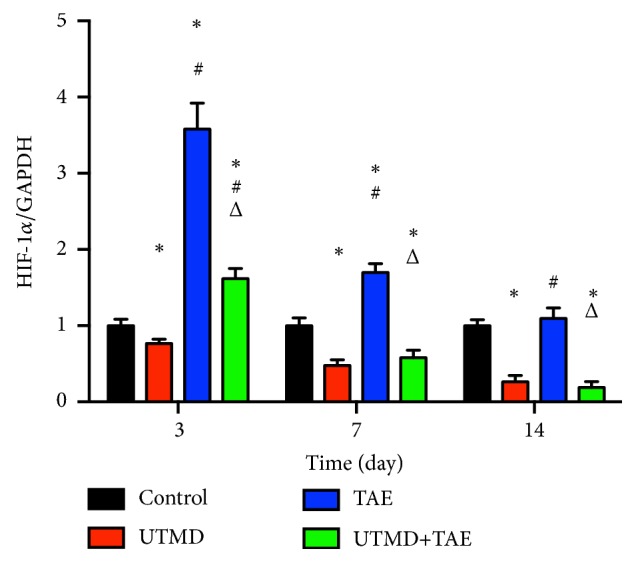
The relative expression of HIF-1*α* in four groups. A: Control group; B: UTMD group; C: TAE group; D: UTMD+TAE group. The comparison between two groups at the same time, *∗*p<0.05: comparison with control group; #p<0.05: comparison with UTMD group; Δp<0.05: comparison with TAE group (n=3).

**Figure 6:**
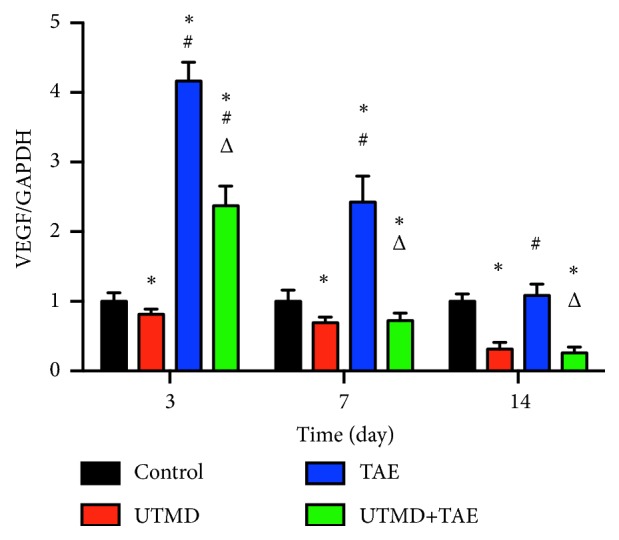
The relative expression of VEGF in four groups. A: Control group; B: UTMD group; C: TAE group; D: UTMD+TAE group. The comparison between two groups at the same time, *∗*p<0.05: comparison with control group; #p<0.05: comparison with UTMD group; Δp<0.05: comparison with TAE group (n=3).

**Figure 7:**
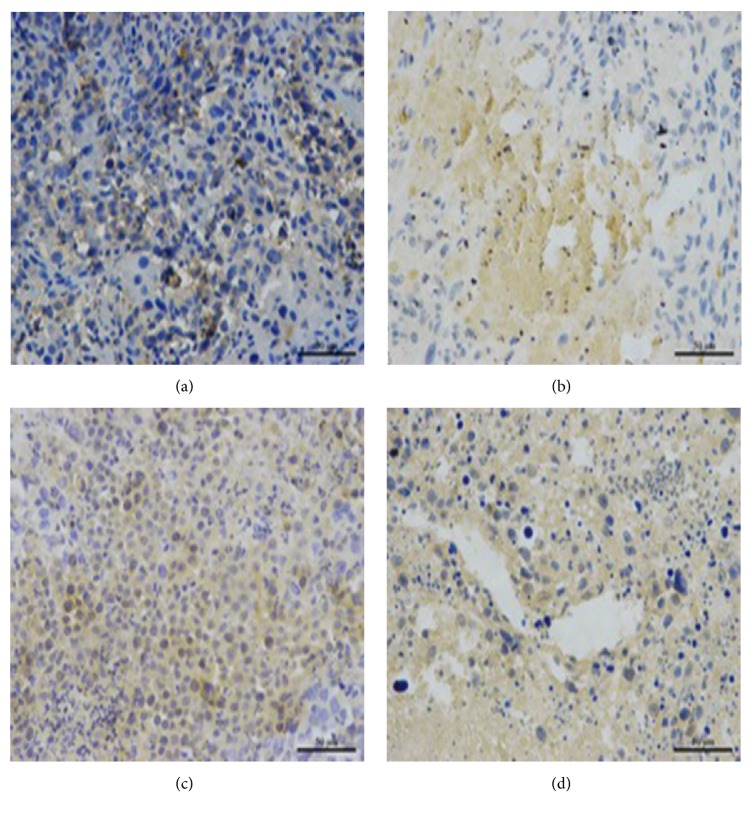
The HIF-1*α* protein expression of hepatoma tissue in the four groups on day 14 (×400 folds). (a) Control group; (b) UTMD group; (c) TAE group; (d) UTMD+TAE group.

**Figure 8:**
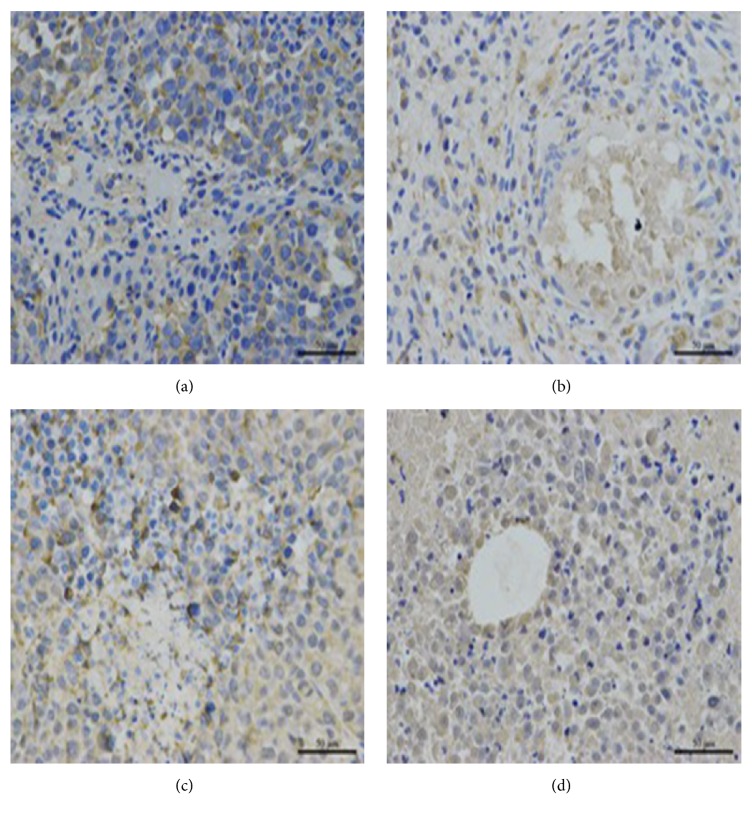
The VEGF protein expression of hepatoma tissue in the four groups on day 14 (×400 folds). (a) Control group; (b) UTMD group; (c) TAE group; (d) UTMD+TAE group.

To compare the protein expression of HIF-1*α* and VEGF in tumor tissue, western blot and immunohistochemical staining were both carried out in the four groups in Figures 4–8. On day 7 and 14, the relative expression of HIF-1*α* was significantly lower in UTMD+TAE group, with a ratio of (0.58±0.10 and 0.19±0.08), compared with the control group (1.00±0.09 and 1.00±0.07), with TAE group (1.70±0.12 and 1.10±0.14) (p<0.05, n=3, respectively). On days 7 and 14, there was no statistical difference between UTMD+TAE group and UTMD group (the ratio of 0.48±0.07 and 0.26±0.08) (p>0.05, n=3). Being similar to HIF-1*α*, there was no statistical difference between UTMD+TAE group and UTMD group of the protein expression of VEGF (p>0.05, n=3). In comparison with UTMD+TAE group (a ratio of 0.72±0.11 and 0.26±0.08), the protein expression of VEGF was obviously higher in the Control group and TAE group (1.00±0.16 and 1.00±0.11 versus 2.43±0.37 and 1.08±0.16) (p<0.05, n=3, respectively). Following paraffin embedding method, the hepatoma tissue was used for immunohistochemical staining. The above change in the four groups was confirmed again in Figures 7 and 8.

## 4. Discussion

In the treatment of liver cancer, whether cancer cells on the edge are death or not is the most important factor that affects the long-term curative effects of the treatment. TAE is not typically the first choice for clinical treatment of liver cancer because it cannot completely kill the cancer cells. Due to this reason, it is usually administered to patients with liver cancer in the middle to late stages where surgery might be too difficult [16]. A prospective trial of TAE for HCC was carried out by Yamashita et al. [17]. In this trial, patients (in different stages) were treated with different methods. They proved that treatment method was the most important factor for therapeutic effect. Currently, there are multiple two way combinations for cancer treatment, such as surgery combined chemotherapy or (and) radiotherapy. This is used to ensure that there is minimal to no tumor recurrence and metastasis. One of the most common combination therapies is TACE [18]. HIF-1*α* is the important factor that influences the treatment effect, and the low expression of HIF-1*α* is very important for preventing tumor recurrence and metastasis after TACE. After the expression of HIF-1*α* mRNA was silenced, cancer cells are in a state of low oxygen and metabolism, as so to the death of cancer [19, 20]. Chen et al. [13] used lentiviral vector to convey the RNAi of HIF-1*α* to McA RH7777 cell. Results proved that it could significantly reduce the expression of HIF-1*α* and VEGF and also inhibit the growth of tumors. In our study, we delivered the HIF-1*α* shRNA into the liver cancer cells, to inhibit blood vessels and the growth of the tumor.

As we all know, the common gene transfection methods (such as virus and liposome) are not applicable for in vivo gene transfection. Virus-mediated gene transfection has the potential of side effects, and the efficiency of liposome-mediated transfection is extremely low in vivo. Presently, there is plenty of research that confirms that UTMD can effectively mediate gene transfection and has many advantages (such as safety, practicality, and target), making it one of the major trends in gene therapy [21–26]. Tang et al. [27] delivered a suicide gene into the hepatic cancer tissue by UTMD. The result showed that the tumor inhibition rate in the treatment group was markedly higher compared with that in the control group (p<0.05). Zhou et al. [28] explored the antitumor effect of UTMD-mediated herpes simplex virus thymidine kinase (HSV-TK) suicide gene system on the models of subcutaneous transplantation tumors. Their results proved that the TK protein expression and tumor inhibitory effect in HSV-TK+MB+US groups were significantly higher than those in other groups (p<0.05). Then it can significantly improve the survival time of tumor-bearing mice. In our study, after the combination treatment between UTMD and TAE, the tumor volume shrank markedly and the survival time was significantly prolonged. It may be caused by blood supply blocking. Through the results of western blotting and immunohistochemical staining, we also found that the protein expression of HIF-1*α* and VEGF in hepatoma tissue greatly reduced. Cancer cells could not survive without good blood supply. However, the mean survival time in the UTMD+TAE group was a little shorter than what was measured in the UTMD group. We suspect that the combination therapy may aggravate the damage to the rats and to determine if this is true, we believe it deserves our further study. In short, there are still difficulties that are needed to overcome in further research (such as target, safety, and high-efficiency) [29, 30].

## Data Availability

The data used to support the findings of this study are available from the corresponding author upon request.

## Abbreviations

HIF-1*α* shRNA: Hypoxia-inducible factor-1*α* short hairpin RNA
UTMD: Ultrasound targeted microbubble destruction
TAE: Transcatheter arterial embolization
CEUS: Contrast-enhanced ultrasound
TACE: Transcatheter arterial chemoembolization
HCC: Hepatocellular carcinoma
IACUC: Institutional Animal Care and Use Committee
TBST: Tris-buffered saline
HSV-TK: Herpes simplex virus thymidine kinase.
